# PASSIM – an open source software system for managing information in biomedical studies

**DOI:** 10.1186/1471-2105-8-52

**Published:** 2007-02-09

**Authors:** Juris Viksna, Edgars Celms, Martins Opmanis, Karlis Podnieks, Peteris Rucevskis, Andris Zarins, Amy Barrett, Sudeshna Guha Neogi, Maria Krestyaninova, Mark I McCarthy, Alvis Brazma, Ugis Sarkans

**Affiliations:** 1 Institute of Mathematics and Computer Science, Riga, University of Latvia, Latvia; 2 European Bioinformatics Institute, EMBL-EBI, Hinxton, UK; 3 Oxford Centre for Diabetes, Endocrinology and Metabolism, Churchill Hospital, Old Road, Headington, Oxford, OX3 7LJ, UK

## Abstract

**Background:**

One of the crucial aspects of day-to-day laboratory information management is collection, storage and retrieval of information about research subjects and biomedical samples. An efficient link between sample data and experiment results is absolutely imperative for a successful outcome of a biomedical study. Currently available software solutions are largely limited to large-scale, expensive commercial Laboratory Information Management Systems (LIMS). Acquiring such LIMS indeed can bring laboratory information management to a higher level, but often implies sufficient investment of time, effort and funds, which are not always available. There is a clear need for lightweight open source systems for patient and sample information management.

**Results:**

We present a web-based tool for submission, management and retrieval of sample and research subject data. The system secures confidentiality by separating anonymized sample information from individuals' records. It is simple and generic, and can be customised for various biomedical studies. Information can be both entered and accessed using the same web interface. User groups and their privileges can be defined. The system is open-source and is supplied with an on-line tutorial and necessary documentation. It has proven to be successful in a large international collaborative project.

**Conclusion:**

The presented system closes the gap between the need and the availability of lightweight software solutions for managing information in biomedical studies involving human research subjects.

## Background

Recording detailed information on collection, processing and storage of samples is crucial both for efficient reporting on any biomedical study and for subsequent data analysis [1]. Collecting and storing this information in a systematic way is particularly important in the context of high-throughput applications, such as proteomics and genomics technologies. Thus, systems facilitating patient and sample data management are in high demand.

We have developed an open-source software system for recording, storing, and providing access to information on biosamples. This system – **P**atient **a**nd **S**ample **S**ystem for **I**nformation **M**anagement (PASSIM) – allows researchers to track information pertinent to sample collection, processing, location, transportation and storage conditions. PASSIM provides an efficient solution to confidentiality issues by separate storage of non-identifiable sample information and records of research participants. The system is web-based, which means that non-identifiable information is kept on a server and can be securely accessed on-line for queries or new submissions by authorised users via web-browser. PASSIM is simple and generic, and thus can be customized for various types of biological studies.

It is worth noting that several publicly available systems include sample-related information in their data models (MiMiR [2], MIMAS [3], ArrayExpress [4]) in order to deepen the integration of sample and experiment data. These data models work well within specific domains (mostly for microarray analysis), but do not allow for effective analysis, integrating various "-omics" data. In principle it might be possible to generalise one of such systems for other types of high-throughput data, however that would further complicate what is already a complex system. We believe that to make such a system more simple and generic, the module used for storage of experiment metadata and results should be separate from the one for the sample information, though they should be interoperable. To the best of our knowledge very few systems of this type are publicly available, e.g. caTISSUE, Open Infrastructure for Outcomes (OIO) [5-7].

The system we present here is a generic version of a system developed for an international collaborative project – Molecular Phenotyping to Accelerate Genomic Epidemiology (MolPAGE). MolPAGE includes 18 academic institutions, biotechnology and pharmaceutical companies (see [8]). In this paper we briefly describe the design principles and the functionality of PASSIM and discuss how the biomedical community can benefit from using such a system and learn from our experience.

## Implementation

### General structure

PASSIM has two main modes:

1) Data submission – entering and editing the information on samples and individuals;

2) Data access – browsing and querying this information, and generating reports.

The submission form is concise, many of the parameters can be reused in a vast spectrum of studies, and more specific ones can be modified or added to the form. At the same time, PASSIM also supports the retrieval of the information, thus representing an effective means of communication and data transfer between sample collection sites and experimentalists.

To deal with the conflicting needs of local researchers (who might wish to retain linkage to non-anonymized subject IDs e.g. as part of ongoing studies) whilst avoiding the potential breaches of security associated with making such data available over the web, we adopted a two-tier solution, consisting of two subsystems:

1) Stand-alone Person Management Tool (PMT), used on-site by the staff collecting the samples [see Additional file 1];

2) Sample Management Database (Sample DB) accessible through the web-based interface [see Additional file 2, figure 1]

**Figure 1:**
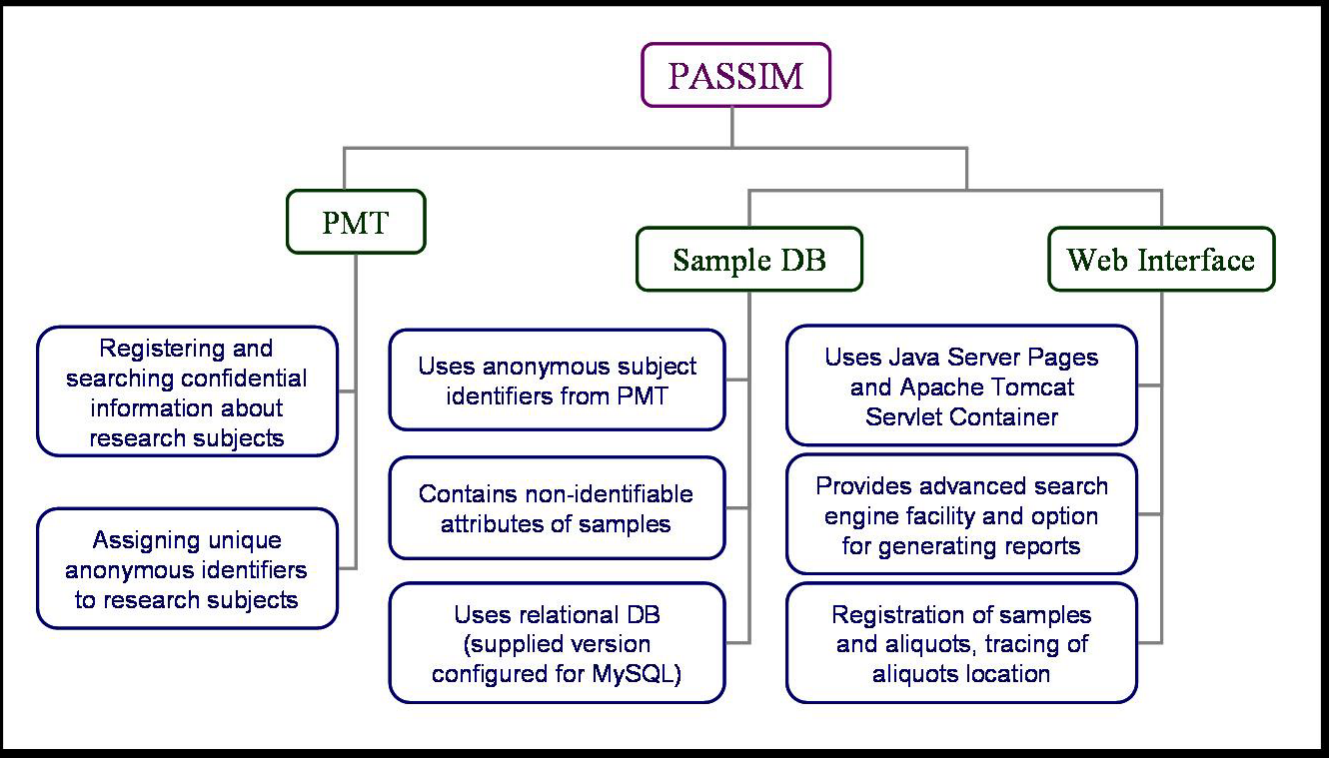
The general structure of PASSIM.

PMT is intended for registering confidential information about the research subjects from whom samples have been taken. As already mentioned, the system assigns a unique anonymous identifier to each individual, which is then used for the individual identification in the Sample DB. Each sample collection site hosts its local copy of the PMT. It is worth noting that keeping identifiable information separately from de-identified information might not be a suitable solution for the studies that require inclusion of identifiable private information into the accessible dataset.

**Figure 2:**
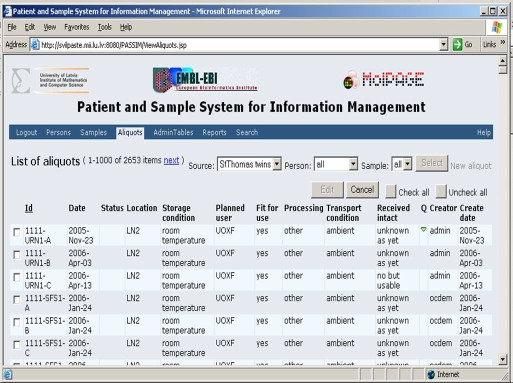
The Sample Database interface.

The Sample DB is accessible online through a web-based interface (using the Java Server Pages technology, and the Apache Tomcat servlet container) and contains non-confidential information about samples. It allows registration of samples and aliquots as well as the subsequent tracing of aliquot locations (see Figure 2). The system is built so that it can work with any traditional relational databases; it was tested on Oracle or MySQL DBMS.

### Object model

**Figure 3:**
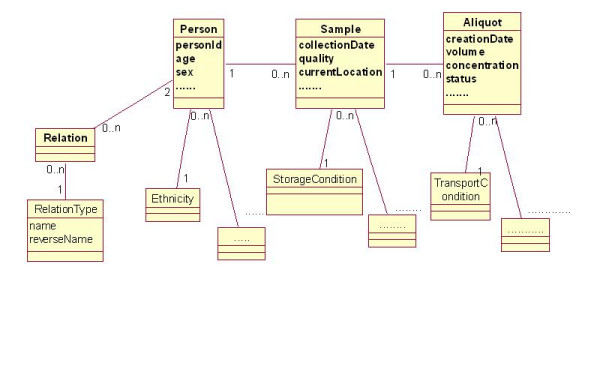
**Central classes and some attributes of the sample management system**. Each sample in the database is associated with only one person; similarly each aliquot is associated with only one sample. There can be an arbitrary number of samples per person and an arbitrary number of aliquots per sample. The total number of tables is 22.

Sample management system is designed around three main classes: PERSONS, SAMPLES and ALIQUOTS (see Figure 3).

All descriptions are entered using controlled vocabularies. Relations (such as "parent", "sibling" etc) between persons are modelled with two additional tables RELATIONS and RELATION_TYPES (Figure 3) and allow storage of an arbitrary number of relations for each person. Details on the storage and transport conditions and on the sample state at its reception are shared between samples and aliquots.

In addition to the three main tables, there is a table USERS that contains information about the system users and their access rights. Table '**Users**' contains information about all the users who can log into the system. Fields **Login name **and **User password **contain the information for logging into the system and should not be empty. Passwords are stored in the database in unencrypted form available to administrator for editing or reminding a forgotten password to a user. There are 4 types of access rights: view-only, access for editing by individual or group users and full access; with an option of editing the administrative tables. For details on the differentiation of the access rights, please see Additional file 1: Table 1. PASSIM is designed for collaborating groups which collect samples in a number of locations and then send them to a different location for analysis. The system allows to register and trace these samples and each group of users has appropriate access to information that is relevant for the group.

**Table 1:** User rights.

**P/S**	**Type**	**Description**
V	*view only access*	All tables can be viewed; no changes are allowed
O	*user data access*	All tables can be viewed. Can add new entries to the database. Editing and deleting is allowed for all entries.
G	*group data access*	All tables can be viewed and new person entries can be added. Editing and deleting is allowed for all entries, not only for data entered by user. Also new samples/aliquots can be created only to persons/samples entered by user from the same partner.
A	*full access*	All tables can be viewed and all data can be added/deleted/edited.
0	*No administrative access*	Suffix "0" denotes that the user doesn't have the access to Administrative tables page
1	*administrative access*	Suffix "1" denotes that the user has access to Administrative tables page

The field 'User rights' contains the information about the users access rights and should contain one of the following values: A0, A1, G0, G1, O1, O1, V0, V1. Prefixes "V" "O" "G" "A" and suffixes "0" "1" stand for various types of access rights, as specified in the table.

### Functionality and customisability

Similarly to the object model, web interface design of Sample DB is based on three main pages: "Persons", "Samples" and "Aliquots", where the corresponding information can be entered, edited or deleted. There is an option for batch submission of several aliquots of the same sample or of samples taken from the same subject. The properties of an existing aliquot entry can be transferred and assigned to a newly created aliquot entry. Such "submitter-friendly" design makes the submission process easy as well as decreases the possibility of mistakes coming from retyping the same information. There is also a possibility to edit the same parameter for many aliquots simultaneously, which may help coordinate transportation and storage of samples across locations.

In addition to submission capabilities, the system provides advanced search engine capabilities. The data can be filtered by such properties as date of birth, gender, source, type, disease state, location and storage conditions. For complex queries there is an option of generating a report using a pre-downloaded copy of the Sample Management Database.

Another important feature of PASSIM is that potential values of the parameters specific to aliquots, samples or research subjects can be changed via the same web interface. Thus, the metadata terms (pre-defined vocabularies) can always be adjusted to fit a preferred ontology or controlled vocabulary by users with sufficient access rights. Additional file 2: Table 2 provides more detailed information on what properties for each parameter are editable. A user intending to edit a parameter name would need to have administrator access rights (A0 or A1). Addition of more parameters or metadata terms can be done, but require direct modification of the database as well as some changes in programs.

**Table 2:** Configuration of web pages.

**Field Name**	**Description**
Short name	The column name shown on List of persons/sample/aliquots page
Long name	The column name shown on Edit page
View column number	Column position on **List of persons/sample/aliquot **page. Value "0" means that the property is not shown. Column with a larger **View column number **is displayed to the right from column with smaller (non-zero) number; however, these numbers are not required to be consecutive.
Sort in view	If **true**, data on **List of persons/sample/aliquots **page will be sorted according to the values by clicking on the column name.
Report column number	Column position on **Reports **page.
Show in report	If **true**, the property will be shown in **Reports **page by default.
Sort in report.	If **true**, data on **Reports **page will be sorted according to the values by clicking on the column name.
Filter in report	If **true**, the property will be available in search filter on the **Reports **page.

There are three main web pages displaying the metadata related to Person, Sample and Aliquot, which is governed by three tables – Person metadata Sample metadata and Aliquot metadata respectively. These tables allow to configure fields in the Persons, Samples and Aliquots web pages. There are a number of fields that can be edited for each of the metadata parameters. This table specifies which fields can be edited (left-hand column) and how it will appear on the web interface (right-hand column).

Such a design makes the system flexible towards developing and changing biological vocabularies. Complete guide to the configuration of access rights and web pages is available at the PASSIM website [9].

## Discussion

The initial specifications for the system were developed by the MolPAGE Consortium members. The main aim of MolPAGE is to develop methods to support genomic epidemiology: that is the measurement, manipulation and analysis of "omics"-scale data in large-scale epidemiological samples. The specifications defined a limited number of properties and variables for individuals, samples and aliquots, which were to be recorded. The sample collection took place at 4 collection sites across 3 different countries. The Patient management system, installed at the sample collection sites, was populated with the clinical data. Then, a unique identifier was generated for each patient and this identifier was transferred to the Sample database. This anonymous identifier constituted a basis of the sample and aliquot IDs. The centralised Sample management system was used through a secure web-interface by both the submitters of the sample information and by the partners analysing the samples. The access rights were diversified to meet the needs of various groups of users. The work within the MolPAGE Consortium revealed a few areas for further development of the system, among which were generation of reports, batch uploading and batch editing.

As PASSIM has proven to be successful, we implemented a generic version of this system for a broader scientific community to use it in other biomedical projects of a similar nature. ***Information management support for consistent reporting on biomedical research ***is the rationale behind the creation of PASSIM. This system can potentially assist in a wide range of studies, in which the results cannot be interpreted accurately without sufficient sample information, such as studies of genetic or plasma biomarkers. LIMS systems are conventionally designed to capture the experimental routine from sample collection to data analysis, and these systems are often not the optimal ones to be used specifically for sample-related data and metadata. PASSIM, on the other hand, is a much lighter software solution than LIMS, designed for capturing, storing and browsing sample-related metadata.

Apart from expanded functionality, the application of PASSIM in the MolPAGE project had another important outcome – an object model, which can serve as a basis for a simple home-made relational database, or as a model for standardized data exchange format.

Standardization of reporting on the results is important in many biomedical studies, for instance in epidemiological studies. It imposes new requirements on day-to-day routine information management [10], thus calling for an effective means for the capture and retrieval of sample-related data. At the moment, there are a number of initiatives controlling the manner in which an investigator reports on a newly discovered biomarker or a newly developed diagnostic test [11-13]. There are also Clinical Data Interchange Consortium [14] and Clinical Data Architecture of Health Level Seven program [15]. Should scientific journals endorse the standards for reporting on such studies (similarly to how, for example, it has been done for microarray studies [16]), the level of details required for related publications would necessitate utilization of LIMS or similar tools for metadata recording in any biomedical research group. Unfortunately, commercial software solutions are expensive and not every lab can afford such a system. We feel that PASSIM or systems that can be derived from our approach can close this gap. In future, we plan to link it to the system for storage of high-throughput experiment data, which is currently under development.

## Conclusion

The open-source nature of PASSIM means that, first, it is an affordable solution for data management and, second, more importantly, its source code is available for external inspections and modifications. It can be customized for needs of a particular laboratory. To the best of our knowledge, it is the only open-source system of this kind.

## Availability and requirements

The PASSIM system along with supporting information can be obtained on the . The on-line tutorial provides assistance in training of potential users of the system. Installation guide and system information can help set up and customize PASSIM for a particular project.

Both parts of PASSIM – Sample Management Database and Person Management Tool – can be also downloaded from .

***Project name***: **P**atient and **S**ample **S**ystem for **I**nformation **M**anagement (PASSIM)

***Project home page***: 

***Operating system(s)***: platform independent

***Programming language***: Java

***Other requirements***: Tomcat 5.0 or more, JDK 1.4.2 or more, Apache Ant 1.6.5 or more; the supplied version of the system is configured for MySQL, additional jdbc driver is required for different databases.

***License***: open source, non-restricted

***Any restrictions to use by non-academics***: no restrictions

## Abbreviations

**LIMS: **Laboratory Information Management Systems

**PASSIM: **Patient and Sample System for Information Management

**MolPAGE: **Molecular Phenotyping to Accelerate Genomic Epidemiology

**ID: **identifier

**PMT: **Person Management Tool

**Sample DB: **Sample management Database

## Authors' contributions

JV and KP designed system and DB architecture. MO designed and implemented PMT. PR designed web interface and reports' generation. AZ and EC did Java programming. AB tested the usability of the system. SGN participated in testing, designed the project website and produced the on-line tutorial. MK participated in requirement analysis, wrote the manuscript, and performed technical coordination. MIM and AB did the conceptual design of the system. US participated in conceptual design, performed overall supervision and coordination. All authors read and approved the final manuscript.

## Supplementary Material

Additional File 1 Standalone Person Management Tool (PMT). PMT is intended for registering confidential information about the research subjects from whom samples have been taken. .zip file contains Windows executable, .xml database file and installation description in a README file. Click here for file

Additional File 2 Sample management database. .zip contains sql version of the database, documentation and the files necessary for the installation of the system. Click here for file
