# Supplementary material for: PASSIM – an open source software system for managing information in biomedical studies
Source: BMC Bioinformatics. 2007 Feb 9;8:52. doi: 10.1186/1471-2105-8-52 (PMC1803798; doi:10.1186/1471-2105-8-52)
Supplement: Additional File 2 — Sample management database. .zip contains sql version of the database, documentation and the files necessary for the installation of the system. [file 1471-2105-8-52-S2.zip › Installation/src/web/help_administrative.html]

Help Patient Sample Management System


  

|  |  |
| --- | --- |
|  |  |

  

| Administrative tables help page |
| --- |

  
Well, if you are here, you probably are system administrator and should know what you are doing :)

|  |  |
| --- | --- |
|  |  |
