# Supplementary material for: PASSIM – an open source software system for managing information in biomedical studies
Source: BMC Bioinformatics. 2007 Feb 9;8:52. doi: 10.1186/1471-2105-8-52 (PMC1803798; doi:10.1186/1471-2105-8-52)
Supplement: Additional File 2 — Sample management database. .zip contains sql version of the database, documentation and the files necessary for the installation of the system. [file 1471-2105-8-52-S2.zip › Installation/src/web/help_aliquots.html]

Help Patient Sample Management System


  

|  |  |
| --- | --- |
|  |  |

  

| Aliquots help page |
| --- |

  
**List of aliquots** page allows to view/edit/delete data either for all aliquots currently in database, aliquots
attributed to particular data source, aliquots attributed to a particular person, or aliquots attributed to particular sample.
  
  
- To add a new aliquot to database click on *New aliquot* link at top right corner. To be able to do this you
must be viewing samples attributed to one particular sample and new aliquot will be defined for that sample. This
includes also the option of simultaneous adding of several aliquots (see below).
  
- To edit data about an aliquot click on the corresponding *edit* link.
  
- To delete entry about a particular aliquot click on the corresponding "edit" link and then press select *Delete* button.
  
  
**Add aliquot** and **Edit aliquot** dialog allows you correspondingly to enter data about a new aliquot or to edit data for
already existing aliquot.
  
  
The field **Aliquot Id** in current version is not editable and is assigned automatically. The
id will look like this:
  
  
*"Sample Id" + "-" + "string of letters (starting from A) corresponding to the current number of aliquots belonging to this sample"*.
  
  
All Aliquot Ids attributed to a particular sample will be unique.
  
  
**Processing** field: a protocol number (from A to Z). (If somebody will provide it, we will put here more information about this.)
  
  
**Status** field: a memo field. (We also don't have more precise information about it's use.)
  
  
  
**Quality(select picture)**: The option is intended to store image files characterizing the quality of RNA samples.
Technically you can store any image file that is recongized as such by web browsers. Files with ".gif", ".jpg" and ".bmp"
postfixes and conforming to these formats generally should be supported by most of the browsers. Also, if you are using
*Netscape* browser you will be able to view the image only after exiting the dialog and re-entering it.
  
  
 The meaning of other fields hopefully should be self-apparent.
  
  
There are two options available from **Add aliquot** dialog, if you wish to add several aliquots with the same characteristics:
  
  
- After defining the aliquot press *Add M* button. The currently entered aliquot will be added to database and
you will be re-directed to another
**Add aliquot** dialog containing another aliquot with the properties you entered for previous one (but, of course, with new Aliquot Id).
  
- After defining the aliquot press *Add N* button, after selecting a number (2-20) from the list. The specified number of aliquots
with the same properties (apart from Aliquot Ids) will be added to database.
  
  
Links to other help pages:
  
  
Login help page
  
Persons help page
  
Samples help page
  
Aliquots help page
  
Search help page
  
Reports help page
  
  
The supported browsers are *Internet Explorer* and *Netscape*. Other web browsers might work, but generally
are not tested.
  

|  |  |
| --- | --- |
|  |  |
