# Supplementary material for: PASSIM – an open source software system for managing information in biomedical studies
Source: BMC Bioinformatics. 2007 Feb 9;8:52. doi: 10.1186/1471-2105-8-52 (PMC1803798; doi:10.1186/1471-2105-8-52)
Supplement: Additional File 2 — Sample management database. .zip contains sql version of the database, documentation and the files necessary for the installation of the system. [file 1471-2105-8-52-S2.zip › Installation/src/web/help_login.html]

Help Patient Sample Management System


  

|  |  |
| --- | --- |
|  |  |

  

| Login help page |
| --- |

  
To access sample database you need a valid login name and a password. If you think that you should have access
to this page, but don't have login name/password you can contact the current system administrator (see below).
  
  
Different users might have different access rights to sample database. Current options include:
  
  
- *View only access*. All tables can be viewed, no changes are allowed.
  
- *User data access*. All tables can be viewed and new person entries added. Editing and deleting is possible only
for data entered by this user. Also new samples/aliquots can be created only to persons/samples entered by this user.
  
- *Group data access*. All tables can be viewed and new person entries added. Editing and deleting is possible only
for data entered by user from the same MolPAGE partner. Also new samples/aliquots can be created only to persons/samples
entered by user from the same MolPAGE partner.
  
- *Full access*. All tables can be viewed and all data added/deleted/edited.
  
  
In addition *Administrator tables* page is available for viewing/editing only to users with specific administrator rights.
  
  
Links to other help pages:
  
  
Login help page
  
Persons help page
  
Samples help page
  
Aliquots help page
  
Search help page
  
Reports help page
  
  
The supported browsers are *Internet Explorer* and *Netscape*. Other web browsers might work, but generally
are not tested.
  

|  |  |
| --- | --- |
|  |  |
