# Supplementary material for: PASSIM – an open source software system for managing information in biomedical studies
Source: BMC Bioinformatics. 2007 Feb 9;8:52. doi: 10.1186/1471-2105-8-52 (PMC1803798; doi:10.1186/1471-2105-8-52)
Supplement: Additional File 2 — Sample management database. .zip contains sql version of the database, documentation and the files necessary for the installation of the system. [file 1471-2105-8-52-S2.zip › Installation/src/web/help_persons.html]

Help Patient Sample Management System


  

|  |  |
| --- | --- |
|  |  |

  

| Persons help page |
| --- |

  
**List of persons** page allows to view/edit/delete data either for all persons currently in database, or persons
attributed to particular data source.
  
  
- To add a new person to database click on *New person* link at top right corner.
  
- To edit data about a particular person click on the corresponding *edit* link.
  
- To delete entry about a particular person click on the corresponding *edit* link and then press select *Delete* button.
This option will be available only for persons for which there are no samples in database. If this is not the case,
delete all sample entries first.
  
- To access samples defined for a particular person click on the corresponding *samples* link.
  
  
**Add person** and **Edit person** dialog allows you correspondingly to enter data about a new person or to edit data for already existing person.
  
  
**Person Id** is a required field, which should be unique for each person. The agreement is that this should be in format
  
  
*"MolPAGE partner specific prefix" + "4 digit id" + "-" + "1 control digit"*,
  
  
 although technically the database will accept a wider range of values. As a rule, the person id is
supposed to be generated by "MolPAGE Person Management" tool, and, before adding the entries to database
you should contact the current system administrator to obtain this tool.
  
  
**Relative** fields allow you to define relations ("parent", "child", "twin" etc) between persons. This is possible only
between persons attributed to the same data source, the possible canditates for relatives can be selected from a list.
  
As many relatives as you wish can be defined, but to do this, after adding a new Relative you should press "Update"
button and then enter Edit person dialog again. If you chose a person Y as a relative for person X, a reverse
relation is added for person Y (e.g. if you select Y as a "child" of X, X automatically becomes "parent" of Y).
However these relations are strictly binary with no further implicit assumptions. E.g. even if X is defined as a "sibling" of "Y" and
"Y" as a "sibling" of "Z", "X" will not automatically become a sibling of "Z".
Similarly, you are free to define that "X" is a "child" of "Y" and "Y" is a child of "X".
  
To delete a relation, change it to empty field in list of relative candidates.
  
Be careful - if you attribute a person to different data source, all relations for this person will be deleted!
  
  
 The meaning of other fields hopefully should be self-apparent.
  
  
Links to other help pages:
  
  
Login help page
  
Persons help page
  
Samples help page
  
Aliquots help page
  
Search help page
  
Reports help page
  
  
The supported browsers are *Internet Explorer* and *Netscape*. Other web browsers might work, but generally
are not tested.
  

|  |  |
| --- | --- |
|  |  |
