# Supplementary material for: PASSIM – an open source software system for managing information in biomedical studies
Source: BMC Bioinformatics. 2007 Feb 9;8:52. doi: 10.1186/1471-2105-8-52 (PMC1803798; doi:10.1186/1471-2105-8-52)
Supplement: Additional File 2 — Sample management database. .zip contains sql version of the database, documentation and the files necessary for the installation of the system. [file 1471-2105-8-52-S2.zip › Installation/src/web/help_reports.html]

Help Patient Sample Management System


  

|  |  |
| --- | --- |
|  |  |

  

| Reports help page |
| --- |

  
**Reports** page allows to generate reports containing entries about Persons, Samples or Aliquots with the
values of selected fields equal to the values defined in the corresponding filters. It is also possible to specify
which fields for Persons, Samples or Aliquots entries will be displayed in the generated report.
  
  
To generate report, first define the fields that should be displayed and the filters that will be used.
  
  
You can chose which fields will be displayed in report. To do this go to the corresponding "Settings" page and
check/uncheck the required fields.
  
  
To define a filter for either for Persons, Samples or Aliquots press "Search" button. Filters currently can be
defined for the following fields:
  
  
**Persons filter:** Age, Diabetes status, Gender, Ethnicity, Person source
  
**Sample filter:** Sample type, Location, Storage condition, Transport condition, Reception status
  
**Aliquot filter:** Location, Planned user, Fitness for use, Transport condition, Reception status
  
  
To include a particular field in search, select one or more values from the corresponding list (to select multiple
values or deselect already selected ones use Ctrl key + mouse click). Filters are conjunctive, i.e. if a field is included
in search, database entries satisfying filter values for this field **AND** filter values for all other
fields will be found. If no values for a field are selected, the field is not included in search (i.e. the result is equal to that of search
with all values for this field included in filter, however the search process is more efficient).
Person filter applies also to Sample/Aliquot reports, i.e. only samples aliquots for Persons satisfying Person filter will
be shown. Similarly, Sample filter affects also Aliquot reports.
  
  
After the defining a filter return to report page by pressing "OK" (or "Cancel", if you do not wish to keep changes)
button. To obtain report conforming to the selected filters press the required of "Persons/Samples/Aliquots" buttons.
  
  
**Disclaimer.** Reports page is mainly intended for creating of reports. The process involves downloading the entire content
of the database, followed by subsequential processing on user machine. As a result the process can be comparatively
slow; although technically Reports page might offer more advanced browsing options than Persons/Samples/Aliquots pages,
it is not recommended for database browsing.
  
  
Links to other help pages:
  
  
Login help page
  
Persons help page
  
Samples help page
  
Aliquots help page
  
Search help page
  
Reports help page
  
  
The supported browsers are *Internet Explorer* and *Netscape*. Other web browsers might work, but generally
are not tested.

|  |
| --- |
|  |
|  |

|  |  |
| --- | --- |
|  |  |
