# Supplementary material for: PASSIM – an open source software system for managing information in biomedical studies
Source: BMC Bioinformatics. 2007 Feb 9;8:52. doi: 10.1186/1471-2105-8-52 (PMC1803798; doi:10.1186/1471-2105-8-52)
Supplement: Additional File 2 — Sample management database. .zip contains sql version of the database, documentation and the files necessary for the installation of the system. [file 1471-2105-8-52-S2.zip › Installation/src/web/help_samples.html]

Help Patient Sample Management System


  

|  |  |
| --- | --- |
|  |  |

  

| Samples help page |
| --- |

  
**List of samples** page allows to view/edit/delete data either for all samples currently in database, samples
attributed to particular data source, or samples attributed to particular person.
  
  
- To add a new sample to database click on *New sample* link at top right corner. To be able to do this you
must be viewing samples attributed to one particular person and new sample will be defined for that person.
  
- To edit data about a sample click on the corresponding *edit* link.
  
- To delete entry about a particular sample click on the corresponding "edit" link and then press select *Delete* button.
This option will be available only for samples for which there are no aliquots in database. If this is not the case,
delete all aliquot entries first.
  
- To access aliquots defined for a particular sample click on the corresponding *aliquots* link.
  
  
**Add sample** and **Edit sample** dialog allows you correspondingly to enter data about a new sample or to edit data for already existing sample.
  
  
The field **Sample Id** in current version is not editable and is assigned automatically on the basis of sample type. The
id will look like this:
  
  
*"Person Id (without "-" and control digit)" + "-" + "string of letters, depending from sample type"
+ "the number (starting from 1) of sample belonging to the same parson and having the same sample type"*.
  
  
The prefixes corresponding to sample types are the following:
  
  
Urine: **URN**,
Plasma no PIC - **PLM**,
Plasma PIC - **PPC**,
Serum no PIC - **SRM**,
Serum PIC - **SPC**,
Whole blood PAXgene - **PAX**,
Whole blood DNA - **WBD**,
Differential count - **DIF**,
Fat Abdominal - **FAB**,
Fat Gluteal - **FGL**,
Fat other - **FOT**,
Subcutaneous fat surgical - **SFS**,
Omental fat surgical - **OFS**,
Muscle surgical - **MSS**,
Liver surgical - **LSS**,
Other - **OTH**.
  
  
All Sample Ids attributed to a particular person will be unique.
  
Sample Id will change, if you will change sample type during editing.
  
  
**Processing metadata** field: a protocol number (from 1 to 20) characterizing "information relating to the processing
of the sample after collection and before aliquoting". (If somebody will provide it, we will put here more information about this.)
  
  
**Processing metadata 2** field: a memo field for comments where there are minor deviations from the standard protocol.
  
  
**Processing metadata 3** field: a memo field that can be used for our each partner's own purpose to denote different experimental conditions.
  
  
 The meaning of other fields hopefully should be self-apparent.
  
  
Links to other help pages:
  
  
Login help page
  
Persons help page
  
Samples help page
  
Aliquots help page
  
Search help page
  
Reports help page
  
  
The supported browsers are *Internet Explorer* and *Netscape*. Other web browsers might work, but generally
are not tested.
  

|  |  |
| --- | --- |
|  |  |
