# Supplementary material for: PASSIM – an open source software system for managing information in biomedical studies
Source: BMC Bioinformatics. 2007 Feb 9;8:52. doi: 10.1186/1471-2105-8-52 (PMC1803798; doi:10.1186/1471-2105-8-52)
Supplement: Additional File 2 — Sample management database. .zip contains sql version of the database, documentation and the files necessary for the installation of the system. [file 1471-2105-8-52-S2.zip › Installation/src/web/help_search.html]

Help Patient Sample Management System


  

|  |  |
| --- | --- |
|  |  |

  

| Search help page |
| --- |

  
**Search** page allows to search for Persons/Samples/Aliquots with a values of selected fields equal to the values
defined in search page filters.
  
  
The fields currently available for searching:
  
  
**Persons filter:** Diabetes status, Gender, Ethnicity, Person source
  
**Sample filter:** Sample type, Location, Storage condition, Transport condition, Reception status
  
**Aliquot filter:** Location, Planned user, Fitness for use, Transport condition, Reception status
  
  
To include a particular field in search, select one or more values from the corresponding list (to select multiple
values or deselect already selected ones use Ctrl key + mouse click). Search will find the database entries with
the value of this field equal to one of the selected field values. Filters are conjunctive, i.e. if a field is included
in search, database entries satisfying filter values for this field **AND** filter values for all other
fields will be found.
  
  
If no values for a field are selected, the field is not included in search (i.e. the result is equal to that of search
with all values for this field included in filter, however the search process is more efficient).
  
  
Person filter applies also to Sample/Aliquot searches, i.e. only samples aliquots for Persons satisfying Person filter will
be shown. Similarly, Sample filter affects also Aliquot search.
  
  
After the pressing one of the "Person/Sample/Aliquot search" buttons you will be redirected correspondingly to
Person/Sample/Aliquot pages, where only the entries satisfying the filter values will be displayed. To display
all databse entries press "Reset filter" button.
  
  
Links to other help pages:
  
  
Login help page
  
Persons help page
  
Samples help page
  
Aliquots help page
  
Search help page
  
Reports help page
  
  
The supported browsers are *Internet Explorer* and *Netscape*. Other web browsers might work, but generally
are not tested.

|  |
| --- |
|  |
|  |

|  |  |
| --- | --- |
|  |  |
