# Supplementary figures and images for: PASSIM – an open source software system for managing information in biomedical studies
Source: BMC Bioinformatics. 2007 Feb 9;8:52. doi: 10.1186/1471-2105-8-52 (PMC1803798; doi:10.1186/1471-2105-8-52)

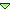

Supplement: Additional File 2 — Sample management database. .zip contains sql version of the database, documentation and the files necessary for the installation of the system. [file 1471-2105-8-52-S2.zip › Installation/src/web/images/down_10.gif]

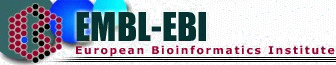

Supplement: Additional File 2 — Sample management database. .zip contains sql version of the database, documentation and the files necessary for the installation of the system. [file 1471-2105-8-52-S2.zip › Installation/src/web/images/ebi.gif]

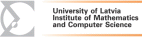

Supplement: Additional File 2 — Sample management database. .zip contains sql version of the database, documentation and the files necessary for the installation of the system. [file 1471-2105-8-52-S2.zip › Installation/src/web/images/imcs.gif]

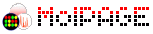

Supplement: Additional File 2 — Sample management database. .zip contains sql version of the database, documentation and the files necessary for the installation of the system. [file 1471-2105-8-52-S2.zip › Installation/src/web/images/molpage.gif]

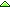

Supplement: Additional File 2 — Sample management database. .zip contains sql version of the database, documentation and the files necessary for the installation of the system. [file 1471-2105-8-52-S2.zip › Installation/src/web/images/up_10.gif]
