# Supplementary material for: PASSIM – an open source software system for managing information in biomedical studies
Source: BMC Bioinformatics. 2007 Feb 9;8:52. doi: 10.1186/1471-2105-8-52 (PMC1803798; doi:10.1186/1471-2105-8-52)
Supplement: Additional File 2 — Sample management database. .zip contains sql version of the database, documentation and the files necessary for the installation of the system. [file 1471-2105-8-52-S2.zip › Documentation/PASSIM_Database_Description.doc]

**Institute of Mathematics and Computer Science**

**European Bioinformatics Institute**

# Description of Sample Management Database

# Version 06.03.2006

**DATABASE SCHEMA**

The current version of the database contains the following tables:

- ALIQUOTS (*)
- ALIQUOT_CONCENTRATION_UNITS
- ALIQUOT_FITNESSES
- ALIQUOT_LOCATION
- ALIQUOT_META_DATA
- ALIQUOT_VOLUME_UNITS
- DISEASE_STATA
- ETHNICITIES
- PARTNERS
- PERSONS (*)
- PERSON_META_DATA
- PERSON_SOURCES
- PROCESSING_OPTIONS
- PROCESSING_PROTOCOLS
- RECEPTION_STATA
- RELATIONS (*)
- RELATION_TYPES
- SAMPLES (*)
- SAMPLE_LOCATIONS
- SAMPLE_META_DATA
- SAMPLE_SUBTYPES
- SAMPLE_TYPES
- SEXES
- STORAGE_CONDITIONS
- TRANSPORT_CONDITIONS
- USERS (*)

From these tables marked with (*): PERSONS, SAMPLES, ALIQUOTS, RELATIONS and USERS represent the dynamic content of the database – entries about persons from which samples are collected, entries about collected samples, entries about aliquots these samples are split into, entries about relations between persons and entries about the current users of database. The remaining tables contain static information about “fixed vocabulary” used to describe various properties of these dynamic entries.

The organization of the main database tables are shown in the Fig. 1 below. For each person in the database (contained in table PERSONS) there can be an arbitrary number of samples (contained in table SAMPLES); each sample belongs to one particular person. Similarly, for each sample in the database there can be an arbitrary number of aliquots (contained in table ALIQUOTS); each aliquot belongs to one particular sample. Relations between persons are stored in table RELATIONS. Each entry in this table references exactly two persons (although technically these can coincide) and the type of relation between them. Also, for each relation defined the system enforces two entries in RELATIONS table – if there is a relation *r* (e.g. *parent*) between persons A and B, then there is also a reverse relation to *x* (e.g., the reverse relation of *parent* is defined as *child*) between persons B and A.

Table USERS have “2 to many” relation with all other database tables, i.e. each entry in these tables has 2 references to table USERS – to the user who has initially created the entry and to the user who has made the last modification to the entry.

Figure 1. Organization of the main database tables.

Tables containing fixed vocabulary entries that are referenced from the tables PERSONS, SAMPLES and ALIQUOTS are correspondingly shown in Fig. 2, Fig. 3 and Fig. 4.

Figure 2. Fixed vocabulary tables referenced from table PERSONS.

Figure 3. Fixed vocabulary tables referenced from table SAMPLES.

Figure 4. Fixed vocabulary tables referenced from table ALIQUOTS.

All these references are of “1 to many” type (except ALIQUOT_VOLUME_UNITS and ALIQUOT_CONCENTRATION_UNITS for which “0..1 to many” type is used), i.e. they are required in corresponding entries in tables PERSONS, SAMPLES in ALIQUOTS. To model situation where the references are unknown at the time when entry is added to the database, all fixed vocabulary tables include entry “unknown” (or semantically similar). Fixed vocabulary tables TRANSPORT_CONDITIONS, STORAGE_CONDITIONS and RECEPTION_STATA are referenced from two tables: SAMPLES and ALIQUOTS; all other are related just to one of the main tables.

Fixed vocabulary describing types of relations are contained in table RELATION_TYPES (Fig. 5). Each relation in table RELATIONS references a particular type of relation from RELATION_TYPES. In addition, each entry from RELATION_TYPES contains a name of reverse relation, which is used by the system to add reverse relation entry in RELATION table.

Figure 5. Fixed vocabulary tables referenced from table RELATIONS.

In addition, there is “0..1 to many” relation between table PARTNERS and USERS, which is used to implement “group access” policy by the system. Each user is assigned to a particular MolPage partner, and, if he is given “group access” rights, is allowed to edit data entered by any of users assigned to the same partner. “0..1” cardinality is used to solve the problems of the initial population of the database.

Figure 6. Relation between tables USERS and PARTNERS.

**GENERAL STRUCTURE OF DATABASE TABLES**

A number of fields are included in all database tables. The information about them is shown in table below. (Fields CREATED_BY and MODIFIED_BY are absent in table USERS.) All FKs used in the database reference SYSID fields in the corresponding tables.

| **Name** | **Datatype** | **Properties** | **Usage** |
| --- | --- | --- | --- |
| SYSID | NUMBER 20,0 | PK | Assigned automatically by system |
| CREATED_BY | NUMBER 20,0 | FK from USERS  Required | Reference to user which created the entry |
| MODIFIED_BY | NUMBER 20,0 | FK from USERS  Required | Reference to user which made the last modification to entry. On creation set to the same value as CREATED_BY |
| CREATED | DATE | Optional | Data and time when the entry was created |
| MODIFIED | DATE | Optional | Data and time when of the last modification of the entry. On creation set to the same value as CREATED |
| COMMENTARY | VARCHAR2 4000 | Optional |  |

The complete list of datatypes used in database tables is given below:

- NUMBER 20,0	Used for SYSID fields and, correspondingly, by FKs
- NUMBER 20,5	Used for numerical values
- DATE	Used to represent years, dates or dates and times
- VARCHAR2 200	Used to represent fixed vocabulary values or short memos
- VARCHAR2 4000	Used for COMMENTARY fields and longer memos

**FIXED VOCABULARY TABLES**

Most of the fixed vocabulary tables (the exceptions are tables RELATION_TYPES, SAMPLE_TYPES and SAMPLE_SUBTYPES that contain some additional fields) contain just one field in addition to the ones described in GENERAL STRUCTURE OF DATABASE TABLES section. This field contains fixed vocabulary terms that represent “semantic meaning” of the references to the table entries. The datatype for all these fields is VARCHAR2 200. Fixed vocabulary terms used in current database version are described in following sections (when describing references to these tables).

| **Table name** | **The name of column containing fixed vocabulary terms** |
| --- | --- |
| ALIQUOT_CONCENTRATION_UNITS | ALIQUOT_CONCENTRATION_UNIT |
| ALIQUOT_FITNESSES | ALIQUOT_FITNESS |
| ALIQUOT_LOCATIONS | ALIQUOT_LOCATION |
| ALIQUOT_VOLUME_UNITS | ALIQUOT_VOLUME_UNIT |
| DISEASE_STATA | DISEASE_STATUS |
| ETHNICITIES | ETHNICITY |
| PARTNERS | PARTNER |
| PERSON_SOURCES | LOCATION_NAME |
| PROCESSING_OPTIONS | PROCESSING_OPTION |
| PROCESSING_PROTOCOLS | PROCESSING_PROTOCOL |
| RECEPTION_STATA | RECEPTION_STATUS |
| RELATION_TYPES | RELATION |
| SAMPLE_LOCATIONS | SAMPLE_LOCATION |
| SAMPLE_SUBTYPES | SAMPLE_SUBTYPE |
| SAMPLE_TYPES | SAMPLE_TYPE |
| SEXES | SEX |
| STORAGE_CONDITIONS | STORAGE_OPTION |
| TRANSPORT_CONDITIONS | TRANSPORT_CONDITIONS |

In addition to this:

- Table RELATION_TYPES contains field REVERSE_RELATION (VARCHAR2 200), containing the name of reverse relation, which is used by the system to add reverse relation entry in RELATION table.
- Table SAMPLE_TYPES contains field ABBREVIATION (VARCHAR2 200), containing sample type dependent postfixes used for automatic generation of sample ids.
- Table SAMPLE_SUBTYPES contains field SAMPLE_TYPE (NUMBER 20,0, FK from SAPLE_TYPES), to reference the sample type to which the given sample subtype belongs.

The current entries in RELATION_TYPES.RELATION and RELATION_TYPES.REVERSE_RELATION fields:

| **RELATION field** | **REVERSE_RELATION field** |
| --- | --- |
| parent | child |
| child | parent |
| sibling | sibling |
| MZ twin | MZ twin |
| DZ twin | DZ twin |
| ?Z twin | ?Z twin |
| spouse | spouse |
| other | other |

The current entries in SAMPLE_TYPES.SAMPLE_TYPE and SAMPLE_TYPES.ABBREVIATION fields:

| **SAMPLE_TYPE field** | **ABBREVIATION field** |
| --- | --- |
| urine | URN |
| plasma no PIC | PLM |
| plasma with PIC | PPC |
| serum no PIC | SRM |
| serum with PIC | SPC |
| whole blood PAXgene | PAX |
| whole blood DNA | WBD |
| differential count | DIF |
| fat biopsy abdominal | FAB |
| fat biopsy gluteal | FGL |
| fat biopsy other | FOT |
| subcutaneous fat sample surgical | SFS |
| omental fat sample surgical | OFS |
| muscle sample surgical | MSS |
| liver sample surgical | LSS |
| other | OTH |

The current entries in SAMPLE_SUBTYPES.SAMPLE_SUBTYPE field for referenced values from SAMPLE_TYPES.SAMPLE_TYPE.

| **SAMPLE_TYPES.SAMPLE_TYPE** | **SAMPLE_SUBTYPES.SAMPLE_SUBTYPE** |
| --- | --- |
| urine | 24 hr Urine  Early morning  Random (spot urine) |
| plasma no PIC | Fasted  Random  Post stimulation  Post prandial  Memo field |
| plasma with PIC | Fasted  Random  Post stimulation  Post prandial  Memo field |
| serum no PIC | Fasted  Random  Post stimulation  Post prandial  Memo field |
| serum with PIC | Fasted  Random  Post stimulation  Post prandial  Memo field |
| whole blood PAXgene | Fasted  Random  Post stimulation  Post prandial  Memo field |
| whole blood DNA | Fasted  Random  Post stimulation  Post prandial  Memo field |
| differential count | Fasted  Random  Post stimulation  Post prandial  Memo field |
| fat biopsy abdominal | Fasted  Random  Post stimulation  Post prandial  Memo field |
| fat biopsy gluteal | Fasted  Random  Post stimulation  Post prandial  Memo field |
| fat biopsy other | Fasted  Random  Post stimulation  Post prandial  Memo field |
| subcutaneous fat sample surgical | Memo only |
| omental fat sample surgical | Memo only |
| muscle sample surgical | Memo only |
| liver sample surgical | Memo only |
| other | other |

**INFORMATION ABOUT PERSONS**

PERSONS table contains the following fields. All FKs refer to SYSID field in referenced table. For tables containing “fixed vocabulary” the column containing referenced values and the values available are shown in “Usage”. Fields described in GENERAL STRUCTURE OF DATABASE TABLES section are omitted here.

| **Name** | **Datatype** | **Properties** | **Usage** |
| --- | --- | --- | --- |
| PERSON_ID | VARCHAR2 200 | Required | Symbol checksum controlled by system  Normally only ids generated by Person Management Tool should be used |
| AGE | DATE | Optional |  |
| SEX | NUMBER 20,0 | FK from SEXES  Required | Referenced values from SEXES.SEX:   - M - F - X |
| ETHNICITY | NUMBER 20,0 | FK from ETHNICITIES  Required | Referenced values from ETHNICITIES.ETHNICITY:   - British/Irish Europid - French Europid - Czech Europid - other European Europid - African origin - South Asian - South East Asian - other |
| ETHNICITY_MEMO | VARCHAR2 200 | Optional | Intended for use only with “other” ethnicity |
| DISEASE_STATUS | NUMBER 20,0 | FK from DISEASE_STATA  Required | Referenced values from DISEASE_STATA.DISEASE_STATUS:   - type 2 diabetes - MODY - type 1 diabetes - other diabetes - prediabetes - normoglyceamic - other |
| DISEASE_MEMO | VARCHAR2 200 | Optional | Intended for use only with “other” diabetes status |
| PERSON_SOURCE | NUMBER 20,0 | FK from PERSON_SOURCES  Required | Referenced values from PERSON_SOURCES.LOCATION_NAME:   - Source 1 - Source 2 - other |
| BMI_1 | NUMBER 20,5 | Optional | Format “*.***” enforced by system |
| YEAR_OF_BMI_1 | DATE | Optional | Only the values for year of DATE are used |
| BMI_2 | NUMBER 20,5 | Optional | Format “*.***” enforced by system |
| YEAR_OF_BMI_2 | DATE | Optional | Only the values for year of DATE are used |
| WHR_1 | NUMBER 20,5 | Optional | Format “***.*” enforced by system |
| YEAR_OF_WHR_1 | DATE | Optional | Only the values for year of DATE are used |
| WHR_2 | NUMBER 20,5 | Optional | Format “***.*” enforced by system |
| YEAR_OF_WHR_2 | DATE | Optional | Only the values for year of DATE are used |

Correspondence between DB tables and fields in Edit Person dialog is shown below. If name of DB table is omitted, table PERSONS is assumed. The names of dialog fields correspond to the entries in table PERSON_META_DATA.

| **Dialog field** | **Table/field** | **Comment** |
| --- | --- | --- |
| Person Id | PERSON_ID | Values controlled by system – see field description |
| DOB | AGE | Should be valid yyyy/mm/dd |
| Diabetes status | DISEASE_STATUS and DISEASE_MEMO | See referenced values in field description |
| Gender | SEX | See referenced values in field description |
| Ethnicity | ETHNICITY and ETHNICITY_MEMO | See referenced values in field description |
| Source | PERSON_SOURCE | See referenced values in field description |
| WHR1 | WHR_1 | Values controlled by system – see field description |
| WHR1/Year | YEAR_OF_WHR_1 | Should be valid yyyy |
| WHR2 | WHR_2 | Values controlled by system – see field description |
| WHR2/Year | YEAR_OF_WHR_2 | Should be valid yyyy |
| BMI1 | BMI_1 | Values controlled by system – see field description |
| BMI1/Year | YEAR_OF_BMI_1 | Should be valid yyyy |
| BMI2 | BMI_2 | Values controlled by system – see field description |
| BMI2/Year | YEAR_OF_BMI_2 | Should be valid yyyy |
| Relative | RELATION_TYPES.RELATION, RELATIONS.PERSON_1,  RELATIONS.PERSON_2,  RELATIONS.RELATION | Referenced values from RELATION_TYPES.RELATION:   - parent - child - sibling - MZ twin - DZ twin - ?Z twin - spouse - other   All information about relatives is stored in RELATIONS table as follows:  RELATIONS.RELATION = RELATION_TYPES.SYSID (corresponding to the selected RELATION_TYPES.RELATION entry);  RELATIONS.PERSON_1 = SYSID (corresponding to the current Edit Dialog);  RELATIONS.PERSON_2 = SYSID (corresponding to the selected person).  A reverse relation entry for PERSON_2 is also stored in RELATIONS table |
| Comment | COMMENTARY |  |

**INFORMATION ABOUT SAMPLES**

SAMPLES table contains the following fields. All FKs refer to SYSID field in referenced table. For tables containing “fixed vocabulary” the column containing referenced values and the values available are shown in “Usage”. Fields described in GENERAL STRUCTURE OF DATABASE TABLES section are omitted here.

| **Name** | **Datatype** | **Properties** | **Usage** |
| --- | --- | --- | --- |
| SAMPLE_ID | VARCHAR2 200 | Required | Assigned automatically by system |
| PERSON | NUMBER 20,0 | FK from PERSONS  Required | Refers to the person from which the sample is collected |
| COLLECTION_DATE | DATE | Optional | Should be valid yyyy/mm/dd |
| SAMPLE_SUBTYPE | NUMBER 20,0 | FK from SAMPLE_SUBTYPES  Required | Referenced values from SAMPLE_SUBTYPES. SAMPLE_SUBTYPE  For complete list of values see FIXED VOCABULARY TABLES section |
| SAMPLE_TYPE_  MEMO | VARCHAR2 200 | Optional | Applicable for all sample subtypes, except “urine” |
| PROCESSING_  METADATA_1 | NUMBER 20,0 | FK from PROCESSING_  PROTOCOLS  Required | Referenced values from PROCESSING_PROTOCOLS.  PROCESSING_PROTOCOL:  Protocol numbers ranging from 1 to 20. The meaning of protocol numbers is defined by users |
| PROCESSING_  METADATA_2 | VARCHAR2 4000 | Optional | Intended to describe deviations from standard protocols |
| PROCESSING_  METADATA_3 | VARCHAR2 4000 | Optional | Additional protocol related information, by users choice |
| STORAGE_AT_  PERIPHERY | NUMBER 20,0 | FK from STORAGE_  CONDITIONS  Required | Referenced values from STORAGE_  CONDITIONS.STORAGE_OPTION:   - room temperature - 4C - -20C - -80C - liquid nitrogen - other |
| CURRENT_  LOCATION | NUMBER 20,0 | FK from SAMPLE_  LOCATIONS  Required | Referenced values from SAMPLE_  LOCATIONS.SAMPLE_LOCATION:   - periphery - central - sent directly to user - other |
| TRANSPORT_  CONDITIONS | NUMBER 20,0 | FK from TRANSPORT_  CONDITIONS  Required | Referenced values from TRANSPORT_  CONDITIONS.TRANSPORT_  CONDITIONS:   - ambient - on ice - on dry ice - other |
| RECEPTION_  STATUS | NUMBER 20,0 | FK from RECEPTION_  STATA  Required | Referenced values from RECEPTION_  STATA.RECEPTION_STATUS:   - unknown as yet - yes - no but usable - no and useless |

Correspondence between DB tables and fields in Edit Sample dialog is shown below. If name of DB table is omitted, table SAMPLES is assumed. The names of dialog fields correspond to the entries in table SAMPLE_META_DATA.

| **Dialog field** | **Table/field** | **Comment** |
| --- | --- | --- |
| Sample Id | SAMPLE_ID | Assigned automatically by system (adding to person id the prefix from SAMPLE_TYPES. ABBREVIATION field followed by number) |
| Collection date | COLLECTION_DATE | Should be valid yyyy/mm/dd/hh/mm |
| Type/Collection metadata | SAMPLE_TYPE, SAMPLE_SUBTYPE and SAMPLE_TYPE_MEMO | Subtypes available for selection depend from the selected sample type. See referenced values in FIXED VOCABULARY TABLES section |
| Location | CURRENT_LOCATION | See referenced values in field description |
| Processing metadata 1 | PROCESSING_METADATA_1 | See referenced values in field description |
| Processing metadata 2 | PROCESSING_METADATA_2 |  |
| Processing metadata 3 | PROCESSING_METADATA_3 |  |
| Storage condition | STORAGE_AT_PERIPHERY | See referenced values in field description |
| Transport-out condition | TRANSPORT_CONDITIONS | See referenced values in field description |
| Received intact | RECEPTION_STATUS | See referenced values in field description |
| Comment | COMMENTARY |  |

**INFORMATION ABOUT ALIQUOTS**

ALIQUOTS table contains the following fields. All FKs refer to SYSID field in referenced table. For tables containing “fixed vocabulary” the column containing referenced values and the values available are shown in “Usage”. Fields described in GENERAL STRUCTURE OF DATABASE TABLES section are omitted here.

| **Name** | **Datatype** | **Properties** | **Usage** |
| --- | --- | --- | --- |
| ALIQUOT_ID | VARCHAR2 200 | Required | Assigned automatically by system |
| FROM_SAMPLE | NUMBER 20,0 | FK from SAMPLES  Required | Refers to the sample from which the aliquot is derived |
| CREATION_DATE | DATE | Optional |  |
| VOLUME | NUMBER 20,5 | Optional | The meaning depends from VOLUME_UNIT that is used |
| CONCENTRATION | NUMBER 20,5 | Optional | The meaning depends from CONCENTRATION_UNIT that is used |
| PROCESSING_  OPTIONS | NUMBER 20,0 | FK from PROCESSING_  PROTOCOLS  Required | Referenced values from PROCESSING_OPTIONS.  PROCESSING_OPTION:  Protocol numbers ranging from A to Z. The meaning of protocol numbers is defined by users |
| STATUS | VARCHAR2 200 | Optional |  |
| STORAGE_  CONDITIONS | NUMBER 20,0 | FK from STORAGE_  CONDITIONS  Required | Referenced values from STORAGE_  CONDITIONS.STORAGE_OPTION:   - room temperature - 4C - -20C - -80C - liquid nitrogen - other |
| STORAGE_  LOCATION | NUMBER 20,0 | FK from ALIQUOT_  LOCATIONS  Required | Referenced values from ALIQUOT_  LOCATIONS.ALIQUOT_LOCATION:   - Location 1 - Location 2 - shipped - other |
| PLANNED_  USER | NUMBER 20,0 | FK from PARTNERS  Required | Referenced values from PARTNERS. PARTNER:   - Partner 1 - Partner 2 - unknown |
| FITNESS_FOR_USE | NUMBER 20,0 | FK from ALIQUOT_  FITNESSES  Required | Referenced values from ALIQUOT_  FITNESSES.ALIQUOT_FITNESS:   - yes - no - other |
| SHIPMENT_DATE | DATE | Optional | - Should be valid yyyy/mm/dd |
| TRANSPORT_  CONDITIONS | NUMBER 20,0 | FK from TRANSPORT_  CONDITIONS  Required | Referenced values from TRANSPORT_  CONDITIONS.TRANSPORT_  CONDITIONS:   - ambient - on ice - on dry ice - other |
| RECEPTION_  STATUS | NUMBER 20,0 | FK from RECEPTION_  STATA  Required | Referenced values from RECEPTION_  STATA.RECEPTION_STATUS:   - unknown as yet - yes - no but usable - no and useless |
| ALIQUOT_  QUALITY_  FILENAME | VARCHAR2 200 | Optional | If present refers to picture file characterizing aliquot quality |
| VOLUME_  UNIT | NUMBER 20,0 | FK from ALIQUOT_  VOLUME_UNITS  Optional | Referenced values from ALIQUOT_  VOLUME_UNITS.ALIQUOT_  VOLUME_UNIT:   - micro l - ml - l - g - kg |
| CONCENTRATION_  UNIT | NUMBER 20,0 | FK from ALIQUOT_  CONCENTRATION_  UNITS  Optional | Referenced values from ALIQUOT_  CONCENTRATION_UNITS. ALIQUOT_CONCENTRATION_  UNIT:   - ng/micro l - micro g/micro l |

Correspondence between DB tables and fields in Edit Sample dialog is shown below. If name of DB table is omitted, table ALIQUOTS is assumed. The names of dialog fields correspond to the entries in table ALIQUOT_META_DATA.

| **Dialog field** | **Table/field** | **Comment** |
| --- | --- | --- |
| Aliquot Id | ALIQUOT_ID | Assigned automatically by system (adding a letter to sample id) |
| Aliquot creation date | CREATION_DATE | Should be valid yyyy/mm/dd |
| Location | STORAGE_LOCATION | See referenced values in field description |
| Planned user | PLANNED_USER | See referenced values in field description |
| Fit for use | FITNESS_FOR_USE | See referenced values in field description |
| Processing | PROCESSING_OPTIONS | See referenced values in field description |
| Storage condition | STORAGE_CONDITIONS | See referenced values in field description |
| Transport-out condition | TRANSPORT_CONDITIONS | See referenced values in field description |
| Received intact | RECEPTION_STATUS | See referenced values in field description |
| Shipment-out date | SHIPMENT_DATE | Should be valid yyyy/mm/dd |
| Status | STATUS |  |
| Volume | VOLUME and VOLUME_UNIT | Decimal number followed by unit - see referenced values in field description |
| Concentration | CONCENTRATION and CONCENTRATION_UNIT | Decimal number followed by unit - see referenced values in field description |
| Comment | COMMENTARY |  |
| Quality | ALIQUOT_QUALITY_  FILENAME | Assigned automatically, when user uploads a picture |

**RELATIONS TABLE**

RELATIONS table contains the following fields. All FKs refer to SYSID field in referenced table. For tables containing “fixed vocabulary” the column containing referenced values and the values available are shown in “Usage”. Fields described in GENERAL STRUCTURE OF DATABASE TABLES section are omitted here.

| **Name** | **Datatype** | **Properties** | **Usage** |
| --- | --- | --- | --- |
| PERSON_1 | NUMBER 20,0 | FK from PERSONS  Required |  |
| PERSON_2 | NUMBER 20,0 | FK from PERSONS  Required |  |
| RELATION | NUMBER 20,0 | FK from RELATION_  TYPES  Required | Referenced values from RELATION_  TYPES.RELATION:   - parent - child - sibling - MZ twin - DZ twin - ?Z twin - spouse - other |

**METADATA TABLES**

Tables PERSON_META_DATA, SAMPLE_META_DATA and ALIQUOT_META_DATA contain string values displayed by the system, mainly these refer to the corresponding fields in PERSONS, SAMPLES and ALIQUOTS tables, as well as information about whether the particular field values can be edited, used for sorting or filtering and in which order these fields are to be displayed.

All these tables contain the following fields in addition to the ones described in GENERAL STRUCTURE OF DATABASE TABLES section.

| **Name** | **Datatype** | **Properties** | **Usage** |
| --- | --- | --- | --- |
| PROPERTY_NAME | VARCHAR 200 |  | Name of referenced table field (as  used in Hibernate mappings) |
| SHORT_NAME | VARCHAR 200 |  | String values used in column headers |
| LONG_NAME | VARCHAR 200 |  | String values used in Edit dialogs |
| VIEW_COLUMN_NUMBER | NUMBER 20,0 |  | Display order in pages  Persons/Samples/Aliquots (not displayed for values 0) |
| IS_SORTABLE | NUMBER 20,0 |  | Value 1 for fields by which data can be sorted in pages Persons/Samples/Aliquots, value 0 for others |
| IS_EDITABLE | NUMBER 20,0 |  | Value 1 for fields values of which can be edited, value 0 for others |
| COLUMN_NUMBER_IN  REPORTS | NUMBER 20,0 |  | Display order in page Reports (not displayed for values 0) |
| SHOW_IN_REPORTS | NUMBER 20,0 |  | Value 1 for fields, which are displayed in page Reports by default, value 0 for others |
| SORT_IN_REPORTS | NUMBER 20,0 |  | Value 1 for fields by which data can be sorted in page Reports, value 0 for others |
| FILTER_IN_REPORTS | NUMBER 20,0 |  | Value 1 for fields by which data can be filtered in page Reports, value 0 for others |

**USERS TABLE**

USERS table contains the following fields. All FKs refer to SYSID field in referenced table. For tables containing “fixed vocabulary” the column containing referenced values and the values available are shown in “Usage”. Fields described in GENERAL STRUCTURE OF DATABASE TABLES section are omitted here (fields CREATED_BY and MODIFIED_BY are missing for this table).

| **Name** | **Datatype** | **Properties** | **Usage** |
| --- | --- | --- | --- |
| FULLNAME | VARCHAR2 200 | Optional |  |
| LOGIN | VARCHAR2 200 | Required | All login names should be different |
| PASSWD | VARCHAR2 200 | Required | User password. Currently no restrictions (including the possibility that password is empty string). Saved as clear text and visible to users with administrator rights. |
| RIGHTS | VARCHAR2 200 | Required | Defines user access rights. Currently 2 symbol string. Values for the first symbol: “V”= “view only access”; “O” = “user data access”, “G” – “group data access” and “A” – “full access”. Values for the second symbol: “0” = “no administrator rights”, “1” = “has administrator rights”. There is no practical use to give less than full access rights for user with administrator privileges. |
| PARTNER | NUMBER 20,0 | FK from PARTNERS  Optional | Referenced values from PARTNERS. PARTNER:   - Partner 1 - Partner 2 - unknown |

**OTHER DATABASE OBJECTS**

In addition to tables, the current database version includes the following objects:

- Sequence HIBERNATE_SEQUENCE. Starting value 1000000, increment interval 1. The sequence is used to assign unique SYSID values by the system. Starting value 1000000 is chosen to allow manual insertion/editing of database entries (SYSID values less than 1000000 should be used for such entries).
- Index UNIQUE_SAMPLE_ID. Enforces uniqueness of value pairs (SAMPLES.SAMPLE_ID, SAMPLES.PERSON). Used to guarantee that all ids for samples taken from a particular person will be unique.
- Index UNIQUE_ALIQUOT_IDS. Enforces uniqueness of value pairs (ALIQUOTS.ALIQUOT_ID, ALIQUOTS.FROM_SAMPLE). Used to guarantee that all ids for aliquots derived from a particular sample will be unique.
- Index UNIQUE_SAMPLE_SUBTYPE_ID. Enforces uniqueness of value pairs (SAMPLE_SUBTYPES.SAMPLE_TYPE, SAMPLES_SUBTYPES.SAMPLE_SUBTYPE). Used to guarantee that all subtypes for a given sample type will have unique names.
- Index SYS_C002715_1. Enforces uniqueness of values USERS.LOGIN. Used to guarantee that all users have different login names.
