# Supplementary material for: PASSIM – an open source software system for managing information in biomedical studies
Source: BMC Bioinformatics. 2007 Feb 9;8:52. doi: 10.1186/1471-2105-8-52 (PMC1803798; doi:10.1186/1471-2105-8-52)
Supplement: Additional File 2 — Sample management database. .zip contains sql version of the database, documentation and the files necessary for the installation of the system. [file 1471-2105-8-52-S2.zip › Documentation/PASSIM_User_Guide.doc]

**Patient and Sample System for Information Management**

**Login**

To access sample database you need a valid login name and a password. If you think that you should have access to this page, but don't have login name/password you can contact the current system administrator (see below).

Different users might have different access rights to sample database. Current options include:

- *View only access*. All tables can be viewed, no changes are allowed.
- *User data access*. All tables can be viewed and new person entries added. Editing and deleting is possible only for data entered by this user. Also new samples/aliquots can be created only to persons/samples entered by this user.
- *Group data access*. All tables can be viewed and new person entries added. Editing and deleting is possible only for data entered by user from the same MolPAGE partner. Also new samples/aliquots can be created only to persons/samples entered by user from the same MolPAGE partner.
- *Full access*. All tables can be viewed and all data added/deleted/edited.

In addition *Administrator tables* page is available for viewing/editing only to users with specific administrator rights.


**Persons**

**List of persons** page allows to view/edit/delete data either for all persons currently in database, or persons attributed to particular data source.

- To add a new person to database click on *New person* link at top right corner.
- To edit data about a particular person click on the corresponding *edit* link.
- To delete entry about a particular person click on the corresponding *edit* link and then press select *Delete* button. This option will be available only for persons for which there are no samples in database. If this is not the case, delete all sample entries first.
- To access samples defined for a particular person click on the corresponding *samples* link.

**Add person** and **Edit person** dialog allows you correspondingly to enter data about a new person or to edit data for already existing person.

**Person Id** is a required field, which should be unique for each person. The agreement is that this should be in format

*"MolPAGE partner specific prefix" + "4 digit id" + "-" + "1 control digit"*,

although technically the database will accept a wider range of values. As a rule, the person id is supposed to be generated by "MolPAGE Person Management" tool, and, before adding the entries to database you should contact the current system administrator to obtain this tool.

**Relative** fields allow you to define relations ("parent", "child", "twin" etc) between persons. This is possible only between persons attributed to the same data source, the possible candidates for relatives can be selected from a list.
As many relatives as you wish can be defined, but to do this, after adding a new Relative you should press "Update" button and then enter Edit person dialog again. If you chose a person Y as a relative for person X, a reverse relation is added for person Y (e.g. if you select Y as a "child" of X, X automatically becomes "parent" of Y). However these relations are strictly binary with no further implicit assumptions. E.g. even if X is defined as a "sibling" of "Y" and "Y" as a "sibling" of "Z", "X" will not automatically become a sibling of "Z". Similarly, you are free to define that "X" is a "child" of "Y" and "Y" is a child of "X".
To delete a relation, change it to empty field in list of relative candidates.
Be careful - if you attribute a person to different data source, all relations for this person will be deleted!

The meaning of other fields hopefully should be self-apparent.

 **Samples**

**List of samples** page allows to view/edit/delete data either for all samples currently in database, samples attributed to particular data source, or samples attributed to particular person.

- To add a new sample to database click on *New sample* link at top right corner. To be able to do this you must be viewing samples attributed to one particular person and new sample will be defined for that person.
- To edit data about a sample click on the corresponding *edit* link.
- To delete entry about a particular sample click on the corresponding "edit" link and then press select *Delete* button. This option will be available only for samples for which there are no aliquots in database. If this is not the case, delete all aliquot entries first.
- To access aliquots defined for a particular sample click on the corresponding *aliquots* link.

**Add sample** and **Edit sample** dialog allows you correspondingly to enter data about a new sample or to edit data for already existing sample.

The field **Sample Id** in current version is not editable and is assigned automatically on the basis of sample type. The id will look like this:

*"Person Id (without "-" and control digit)" + "-" + "string of letters, depending from sample type" + "the number (starting from 1) of sample belonging to the same parson and having the same sample type"*.

The prefixes corresponding to sample types are the following:

Urine: **URN**, Plasma no PIC - **PLM**, Plasma PIC - **PPC**, Serum no PIC - **SRM**, Serum PIC - **SPC**, Whole blood PAXgene - **PAX**, Whole blood DNA - **WBD**, Differential count - **DIF**, Fat Abdominal - **FAB**, Fat Gluteal - **FGL**, Fat other - **FOT**, Subcutaneous fat surgical - **SFS**, Omental fat surgical - **OFS**, Muscle surgical - **MSS**, Liver surgical - **LSS**, Other - **OTH**.

All Sample Ids attributed to a particular person will be unique.
Sample Id will change, if you will change sample type during editing.

**Processing metadata** field: a protocol number (from 1 to 20) characterizing "information relating to the processing of the sample after collection and before aliquoting". (If somebody will provide it, we will put here more information about this.)

**Processing metadata 2** field: a memo field for comments where there are minor deviations from the standard protocol.

**Processing metadata 3** field: a memo field that can be used for our each partner's own purpose to denote different experimental conditions.

The meaning of other fields hopefully should be self-apparent.


**Aliquots**

**List of aliquots** page allows to view/edit/delete data either for all aliquots currently in database, aliquots attributed to particular data source, aliquots attributed to a particular person, or aliquots attributed to particular sample.

- To add a new aliquot to database click on *New aliquot* link at top right corner. To be able to do this you must be viewing samples attributed to one particular sample and new aliquot will be defined for that sample. This includes also the option of simultaneous adding of several aliquots (see below).
- To edit data about an aliquot click on the corresponding *edit* link.
- To delete entry about a particular aliquot click on the corresponding "edit" link and then press select *Delete* button.

**Add aliquot** and **Edit aliquot** dialog allows you correspondingly to enter data about a new aliquot or to edit data for already existing aliquot.

The field **Aliquot Id** in current version is not editable and is assigned automatically. The id will look like this:

*"Sample Id" + "-" + "string of letters (starting from A) corresponding to the current number of aliquots belonging to this sample"*.

All Aliquot Ids attributed to a particular sample will be unique.

**Processing** field: a protocol number (from A to Z). (If somebody will provide it, we will put here more information about this.)

**Status** field: a memo field. (We also don't have more precise information about it's use.)


**Quality(select picture)**: The option is intended to store image files characterizing the quality of RNA samples. Technically you can store any image file that is recognized as such by web browsers. Files with ".gif", ".jpg" and ".bmp" postfixes and conforming to these formats generally should be supported by most of the browsers. Also, if you are using *Netscape* browser you will be able to view the image only after exiting the dialog and re-entering it.

The meaning of other fields hopefully should be self-apparent.

There are two options available from **Add aliquot** dialog, if you wish to add several aliquots with the same characteristics:

- After defining the aliquot press *Add M* button. The currently entered aliquot will be added to database and you will be re-directed to another **Add aliquot** dialog containing another aliquot with the properties you entered for previous one (but, of course, with new Aliquot Id).
- After defining the aliquot press *Add N* button, after selecting a number (2-20) from the list. The specified number of aliquots with the same properties (apart from Aliquot Ids) will be added to database.


**Search**

**Search** page allows to search for Persons/Samples/Aliquots with values of selected fields equal to the values defined in search page filters.

The fields currently available for searching:

**Persons filter:** Diabetes status, Gender, Ethnicity, Person source
**Sample filter:** Sample type, Location, Storage condition, Transport condition, Reception status
**Aliquot filter:** Location, Planned user, Fitness for use, Transport condition, Reception status

To include a particular field in search, select one or more values from the corresponding list (to select multiple values or deselect already selected ones use Ctrl key + mouse click). Search will find the database entries with the value of this field equal to one of the selected field values. Filters are conjunctive, i.e. if a field is included in search, database entries satisfying filter values for this field **AND** filter values for all other fields will be found.

If no values for a field are selected, the field is not included in search (i.e. the result is equal to that of search with all values for this field included in filter, however the search process is more efficient).

Person filter applies also to Sample/Aliquot searches, i.e. only samples aliquots for Persons satisfying Person filter will be shown. Similarly, Sample filter affects also Aliquot search.

After the pressing one of the "Person/Sample/Aliquot search" buttons you will be redirected correspondingly to Person/Sample/Aliquot pages, where only the entries satisfying the filter values will be displayed. To display all database entries press "Reset filter" button.

 **Reports**

**Reports** page allows to generate reports containing entries about Persons, Samples or Aliquots with the values of selected fields equal to the values defined in the corresponding filters. It is also possible to specify which fields for Persons, Samples or Aliquots entries will be displayed in the generated report.

To generate report, first define the fields that should be displayed and the filters that will be used.

You can chose which fields will be displayed in report. To do this go to the corresponding "Settings" page and check/uncheck the required fields.

To define a filter for either for Persons, Samples or Aliquots press "Search" button. Filters currently can be defined for the following fields:

**Persons filter:** Age, Diabetes status, Gender, Ethnicity, Person source
**Sample filter:** Sample type, Location, Storage condition, Transport condition, Reception status
**Aliquot filter:** Location, Planned user, Fitness for use, Transport condition, Reception status

To include a particular field in search, select one or more values from the corresponding list (to select multiple values or deselect already selected ones use Ctrl key + mouse click). Filters are conjunctive, i.e. if a field is included in search, database entries satisfying filter values for this field **AND** filter values for all other fields will be found. If no values for a field are selected, the field is not included in search (i.e. the result is equal to that of search with all values for this field included in filter, however the search process is more efficient). Person filter applies also to Sample/Aliquot reports, i.e. only samples aliquots for Persons satisfying Person filter will be shown. Similarly, Sample filter affects also Aliquot reports.

After the defining a filter return to report page by pressing "OK" (or "Cancel", if you do not wish to keep changes) button. To obtain report conforming to the selected filters press the required of "Persons/Samples/Aliquots" buttons.

**Disclaimer.** Reports page is mainly intended for creating of reports. The process involves downloading the entire content of the database, followed by subsequential processing on user machine. As a result the process can be comparatively slow; although technically Reports page might offer more advanced browsing options than Persons/Samples/Aliquots pages, it is not recommended for database browsing.

The supported browsers are *Internet Explorer* and *Netscape*. Other web browsers might work, but generally are not tested.
