# Supplementary material for: PASSIM – an open source software system for managing information in biomedical studies
Source: BMC Bioinformatics. 2007 Feb 9;8:52. doi: 10.1186/1471-2105-8-52 (PMC1803798; doi:10.1186/1471-2105-8-52)
Supplement: Additional File 2 — Sample management database. .zip contains sql version of the database, documentation and the files necessary for the installation of the system. [file 1471-2105-8-52-S2.zip › Documentation/PASSIM_Web_Applications_Description.doc]

**Samples Web Application file description**

Package molpage.com

| ApplicationListener.java | Class to open/close hibernate mappings for entirely web application. Should be included in listener section in configuration file for application (see listener section in web.xml) |
| --- | --- |
| MetadataManager.java | Class to process metadata information |
| FilterObject.java | Class to store runtime database queries (build by DefineFilter.jsp, used by ViewPersons.jsp, ViewSamples.jsp, ViewAliquots.jsp). Destroyed by “cancel filter” operation in view pages. |
| RelationObject.java | Class to store temporary relation information for person (used by AddPerson.jsp and ViewPersons.jsp) |
| RequestParser.java | Class to manage multipart request – necessary for file upload (used by AddAliquotsUpload.jsp) |
| SessionListener.java | Class to support session management for entirely web application. Should be included in listener section in configuration file for application (see listener section in web.xml). |
| UserHttpSess.java | Class to store and manage current session state. Class contains implementation of services for supporting database connectivity (get/insert/delete persistent data). |
| Util.java | Class to support common service tasks for entirely web application. |

Package molpage.hibernate

| Persistent.java | Parent class from which other hibernate java classes (except metadata classes) are derived.  Persistent class contains attributes (id, visibleName, creator, modifier, createDate, modifDate, comment) and appropriate getters, setters. |
| --- | --- |
| Aliquot.hbm.xml | Hibernate mapping file for Aliquots table |
| Aliquot.java | Java class for Aliquots table |
| AliquotConcentrationUnit.hbm.xml | Hibernate mapping file for aliquot_concentration_units table |
| AliquotConcentrationUnit.java | Java class for aliquot_concentration_units table |
| AliquotFitness.hbm.xml | Hibernate mapping file for aliquot_fitnesses table |
| AliquotFitness.java | Java class for aliquot_fitnesses table |
| AliquotLocation.hbm.xml | Hibernate mapping file for aliquot_locations table |
| AliquotLocation.java | Java class for aliquot_locations table |
| AliquotMetaData.hbm.xml | Hibernate mapping file for aliquot_meta_data table |
| AliquotMetaData.java | Java class for aliquot_meta_data table |
| AliquotVolumeUnit.hbm.xml | Hibernate mapping file for aliquot_volume_units table |
| AliquotVolumeUnit.java | Java class for aliquot_volume_units table |
| DiseaseStatus.hbm.xml | Hibernate mapping file for disease_stata table |
| DiseaseStatus.java | Java class for disease_stata table |
| Ethnicity.hbm.xml | Hibernate mapping file for ethnicities table |
| Ethnicity.java | Java class for ethnicities table |
| MetaData.java | Parent class from which other metadata java classes are derived. |
| Partner.hbm.xml | Hibernate mapping file for partners table |
| Partner.java | Java class for partners table |
| Person.hbm.xml | Hibernate mapping file for persons table |
| Person.java | Java class for persons table |
| PersonMetaData.hbm.xml | Hibernate mapping file for person_meta_data table |
| PersonMetaData.java | Java class for person_meta_data table |
| PersonSource.hbm.xml | Hibernate mapping file for person_sources table |
| PersonSource.java | Java class for person_sources table |
| ProcessingOption.hbm.xml | Hibernate mapping file for processing_options table |
| ProcessingOption.java | Java class for processing_options table |
| ProcessingProtocol.hbm.xml | Hibernate mapping file for processing_protocols table |
| ProcessingProtocol.java | Java class for processing_protocols table |
| ReceptionState.hbm.xml | Hibernate mapping file for reception_stata table |
| ReceptionState.java | Java class for reception_stata table |
| Relation.hbm.xml | Hibernate mapping file for relations table |
| Relation.java | Java class for relations table |
| RelationType.hbm.xml | Hibernate mapping file for relation_Types table |
| RelationType.java | Java class for relation_Types table |
| Sample.hbm.xml | Hibernate mapping file for samples table |
| Sample.java | Java class for samples table |
| SampleLocation.hbm.xml | Hibernate mapping file for sample_locations table |
| SampleLocation.java | Java class for sample_locations table |
| SampleMetaData.hbm.xml | Hibernate mapping file for sample_meta_data table |
| SampleMetaData.java | Java class for sample_meta_data table |
| SampleSubType.hbm.xml | Hibernate mapping file for sample_subtypes table |
| SampleSubType.java | Java class for sample_subtypes table |
| SampleType.hbm.xml | Hibernate mapping file for sample_type table |
| SampleType.java | Java class for sample_type table |
| Sex.hbm.xml | Hibernate mapping file for sexes table |
| Sex.java | Java class for sexes table |
| StorageCondition.hbm.xml | Hibernate mapping file for storage_conditions table |
| StorageCondition.java | Java class for storage_conditions table |
| TransportCondition.hbm.xml | Hibernate mapping file for transport_conditions table |
| TransportCondition.java | Java class for transport_conditions table |
| User.hbm.xml | Hibernate mapping file for users table |
| User.java | Java class for users table |

Package molpage.com.xmlWriter

| XMLCreator.java | Java class create report data |
| --- | --- |
| XMLSetting.java | Java class of the setting of the report data |
| XMLUtil.java | Class to support common service tasks for report data |
| XMLWriter.java | Java class create XML file |

Samples Web Application files

| ViewAliquots.jsp | JSP for generating list of aliquots page. Page allows to add/view/edit/delete data either for all aliquots currently in database, aliquots attributed to a particular data source, aliquots attributed to a particular person, or aliquots attributed to a particular sample. |
| --- | --- |
| AddAliquots.jsp | JSP for generating add/edit aliquots page. Page is invoked from ViewAliquots page. |
| AddAliquotsBatch.jsp | JSP for generating add/edit(batch mode) aliquots page. Page allows add/edit multiple aliquots simultaneously. Page is invoked from ViewAliquots page (if batch edit mode is chosen). |
| AddAliquotsUpload.jsp | JSP for generating add/edit aliquots page. Page is invoked from AddAliquots page. Page to manage multipart request (enctype= “multipart/form-data”) – necessary for file upload. |
| AddAliquotsE.jsp | Common part for AddAliquots.jsp, AddAliquotsBatch.jsp, AddAliquotsUpload.jsp. |
| ViewPersons.jsp | JSP for generating list of persons page. Page allows to add/view/edit/delete data either for all persons currently in database, or persons attributed to a particular data source. |
| AddPerson.jsp | JSP for generating add/edit persons page. Page is invoked from ViewPersons page. |
| AddPersonsE.jsp | Auxiliary JSP. JSP is used by AddPerson.jsp. |
| ViewSamples.jsp | JSP for generating list of samples page. Page allows to add/view/edit/delete data either for all samples currently in database, samples attributed to a particular data source, or samples attributed to a particular person. |
| AddSamples.jsp | JSP for generating add/edit samples page. Page is invoked from ViewSamples page. |
| AddSamplesE.jsp | Auxiliary JSP. JSP is used by AddSamples.jsp. |
| AdminPage.jsp | JSP for generating administrator page. Page allows to view data in administrative tables. |
| AdminPageE.jsp | JSP for generating administrator page. Page allows to view/edit/delete data in administrative tables. Page is invoked from AdminPage page. |
| DefineFilter.jsp | JSP for generating DefineFilter page. Page allows to define search parameters for persons, samples, aliquots data. These search parameters are stored in FilterObject.java and used by ViewPersons.jsp, ViewSamples.jsp, ViewAliquots.jsp. |
| Footer.jsp | Included in other JSP files to generate html pages in a uniform way. |
| Header.jsp | Included in other JSP files to generate html pages in a uniform way. |
| LogIn.jsp | JSP for generating login page. To access sample database you need to enter a valid login name and a password. |
| Test_JSP_error.jsp | JSP for generating error page. Page contains error messages. |
| help_administrative.html | Administrative tables help page |
| help_aliquots.html | Aliquots help page |
| help_help.html | Help about help |
| help_login.html | Login help page |
| help_persons.html | Persons help page |
| help_reports.html | Reports help page |
| help_samples.html | Samples help page |
| help_search.html | Search help page |
| formstyle.css | Common style css (Cascading Style Sheets) file. File is used to define how to display HTML elements. |
| utils.js | Java scripts to support common service tasks for entirely web application. |

Image files

| down_10.gif | |  | Picture used in application |
| --- | --- | --- | --- |
| up_10.gif | |  | Picture used in application |
| down_10.gif | |  | Picture used in application |
| favicon.ico | |  | Picture used in application |
| ebi.gif |  | | |
| galva1_2r.gif |  | | |
| imcs.gif |  | | |
| molpage.gif |  | | |

Browser files

| ShowBrowser.jsp | JSP for generating list of samples page. Page allows modify and filter the view of data for all persons, samples and aliquots taken from database. |
| --- | --- |
| htmlContents.js | Java script operate with report HTML file. |

Sample_Qualities folder for alikots qualities (pictures)

Configuration file for Samples Web Application

| web.xml | <web-app>  <context-param>  <param-name>htype</param-name>  <param-value>2</param-value>  </context-param>  <context-param>  <param-name>personid</param-name>  <param-value>check</param-value>  </context-param>  <context-param>  <param-name>viewlimit</param-name>  <param-value>1000</param-value>  </context-param>  <listener>  <listener-class>molpage.com.SessionListener</listener-class>  </listener>  <listener>  <listener-class>molpage.com.ApplicationListener</listener-class>  </listener>  <session-config>  <session-timeout>10</session-timeout>  </session-config>  <welcome-file-list>  <welcome-file>LogIn.jsp</welcome-file>  </welcome-file-list>  <error-page>  <exception-type>java.lang.Exception</exception-type>  <location>/Test_JSP_error.jsp</location>  </error-page>  </web-app> | |
| --- | --- | --- |
| <param-name>htype</param-name>  <param-value>2</param-value> | | 1 – use first header style  2 – use second header style |
| <param-name>personid</param-name>  <param-value>check</param-value> | | check – enable personid checking (if Person Management tool is used)  nocheck – allow arbitrary personids  XXX999 (an arbitrary prefix containing no digits followed by string of “9”-s with the length equal to the desired length of generated part of ids) – generate personids automatically |
| <param-name>viewlimit</param-name>  <param-value>1000</param-value> | | 1000 – number of items in Persons/Samples and Aliquots pages |
| <listener-class>molpage.com.SessionListener</listener-class> | | see SessionListener.java |
| listener-class>molpage.com.ApplicationListener</listener-class> | | see ApplicationListener.java |
| <welcome-file>LogIn.jsp</welcome-file> | | Start page of the application (see LogIn.jsp) |
| <exception-type>java.lang.Exception</exception-type>  <location>/Test_JSP_error.jsp</location> | | Error page for “unexpected” runtime exceptions (see Test_JSP_error.jsp) |
